# Supplementary material for: Are carbon emissions trading and green financial instruments synergistic? -Comprehensive quantitative research based on content analysis
Source: PLoS One. 2024 Mar 7;19(3):e0298601. doi: 10.1371/journal.pone.0298601 (PMC10919601; doi:10.1371/journal.pone.0298601)
Supplement: S1 Data — (ZIP) [file pone.0298601.s001.zip › Data/地级市Z_F工作报告.zip!/║■─╧/│ñ╔│╩╨/└┤╫╘╣1⁄2╓┌║┼í╛┬φ┐╦╩2╛▌═°í┐_2010.docx]

各位代表：

　　现在，我代表市人民政府向大会报告工作，请予审议，并请各位政协委员和其他列席人员提出意见。

　　一、2009年工作回顾

　　2009年是新世纪以来我市经济社会发展最为困难的一年。面对金融危机的严重影响，在市委的坚强领导下，我们科学研判宏观形势，提早谋划重大项目，深入开展“两帮两促”活动，紧紧扭住发展重点和关键环节，坚决而又创造性地贯彻落实党中央、国务院和省委、省政府的一系列决策部署，团结带领全市人民奋力拼搏，攻坚克难，全面完成了市第十三届人民代表大会第二次会议确定的各项目标任务。

　　——实现经济平稳快速增长。全年完成地区生产总值3300亿元，增长14.5 %（预计数，下同）；地方财政收入406亿元，增长18.7 %；全社会固定资产投资2441.8亿元，增长30.3%；社会消费品零售总额1517亿元，增长19.2%；城乡居民人均可支配收入20217元和9005元，分别增长13 %和18 %。

　　——开工建设一批重大项目。紧紧围绕经济社会发展中最紧迫、最急需解决的问题，实施一大批拉动作用大、群众受益大的项目。地铁2号线、过江隧道、湘江综合枢纽工程等重大项目开工建设；武广长沙站及配套工程基本完成，武广高铁正式通车；黄花机场改扩建、职教基地等重大项目顺利推进；广汽菲亚特、比亚迪等一批重大产业项目相继落户。这些重大项目既拉动了当年经济增长，又为长远发展增强了后劲和竞争力。

　　——切实改善民生。把保障和改善民生作为头等大事。全面完成实事工程；全市教育、环境保护、农林水支出分别增长21.03%、66.16%、20.73%；橘子洲公园建成开园，成为长沙的新标志和市民休闲的好去处；城区公园免费向市民开放；取消农村义务教育阶段学校寄宿费，15家游泳池免费向中小学生开放；建成56家社区居家养老服务中心；完成60个农贸市场提质改造。市民的归属感和幸福感不断增强，再次被评为中国十大最具幸福感城市。

　　——着力解决群众住房困难。把安居工程建设作为工作重点，促进房地产市场持续健康发展。全年搬迁棚户区居民1.66万户，拆迁棚户区216.78万平方米，建设(筹集)廉租房7117套，发放经济适用房货币补贴2万余户11.57亿元，1.9万余户低收入居民搬进了新家；商品房销售面积增长71%，房价基本稳定。安居工程成为改善民生、拉动投资、扩大消费的最佳结合点，我市的经验得到中央及有关部门的肯定，全国保障性住房工作会议在长沙召开。

　　——实施一系列城市规划建设管理新举措。紧紧围绕提升城市品质和改善人居环境，推进城市规划建设管理创新。编制实施《城市色彩规划》，着力打造和谐素雅的城市色彩；修订《城市绿线管理办法》，加强城市绿地管理；继续实施交通疏导工程，着力解决交通拥堵问题，支撑了城市车辆保有量由57万辆增加到 75万辆的通行需求；首创群众有奖举报城市管理问题数字化信息平台，市民主动参与城市管理；完成社区环境综合整治40个，建成社区公园6个，荣获联合国“人居环境良好范例奖”。

　　——改进政府管理服务。着力建设服务、效能、阳光政府，提高政府决策透明度和群众参与度。率先全国实行市政府常务会议、记者招待会电视、电台、网络同步直播；行政许可项目在线审批达100%，行政审批时限再缩短30%；取消行政事业性收费项目208项，为群众和企业减轻负担8300万元。着力解决历史遗留问题和各类信访问题，建立领导干部定期集中接访制度，及时处理经济社会发展和城市建设中的矛盾和问题。

　　各位代表！尽管2009年全市经济社会发展遇到的困难大于预期，但我们开展了富有成效的工作，经受住了考验，实现了省委、省政府“弯道超车”和市委“逆势而上、率先发展”的要求。城市综合竞争力跃居全国省会城市前列，4县（市）全部进入全国县域经济基本竞争力百强县（市），对外吸引力不断增强，国内外客商纷至沓来、知名企业纷纷落户，黄花机场进出港达1128万人次。被评为首届全国最具软实力城市、十大创业之城、十大品牌城市，知名度和影响力显著提升。

　　全年经济社会发展主要有以下几个特点：

　　（一）项目建设成为推动经济增长的直接动力。重点实施十大基础设施、十大民生事业、十大产业建设工程，开工建设亿元以上项目38个，322 个重大项目累计完成投资586亿元。启动开元东路、长浏高速等项目建设，中航飞机起落架、陕汽重卡等项目建设进展顺利。加大融资力度，争取国家重大项目投资156.3亿元，引进外资20.33亿美元、增长12.9%，引进市外境内资金575亿元、增长13.2%，发行企业债券、中期票据140亿元，成立先导投资控股有限公司，5家公司在境内外首发上市，保证了大规模建设资金需要。项目建设成为投资快速增长的主要支撑。

　　（二）产业壮大为持续发展注入强劲活力。实施重点产业振兴规划，工业总产值迈上4000亿元台阶，增长23.8%；实现规模以上工业增加值1120亿元，增长19%；高新技术产业产值1440亿元，增长28%；三一、中联、中烟3家企业年销售收入均过300亿元。深化产学研金合作，研发经费占GDP比重2%，被评为国家科技进步示范市，高新区获批国家创新型科技园区试点，经开区获批国家新型工业化产业示范基地。实施知识产权战略，发明专利授权总量居省会和副省级城市第6位。推进质量兴市，新增中国驰名商标4个，总量居中部首位。金霞保税物流中心封关运行，芙蓉中央商务区加快建设，服务外包业务总量增长30.4%，5家物流企业进入全国百强，霞凝港集装箱年吞吐量居全国内河港首位。成功承办第五届两岸经贸文化论坛、首届中部农业博览会等重大节会。推进靖港古镇、灰汤温泉等旅游项目建设，实现旅游总收入355.8亿元。落实家电、汽车下乡及以旧换新政策，举办“福满星城”购物消费节，发放消费券，促进居民消费，消费对经济增长的贡献率达40%。

　　（三）宜居城市建设促进城市品质提升。启动城市总体规划和综合交通规划修编。实施工程建设质量精细化管理“三年行动计划”，“一江两岸”道路建设基本完成，19条道路建成通车，完成车站北路、城南路、人民中路、新民路、东风路等5条特色景观街建设。中山西路、东牌楼等重点棚改项目进展顺利。启动老旧城区社区物业服务试点，拆除户外广告35万平方米，重点整治五一广场等夜市，市容市貌明显改观。完成城市林业生态圈造林任务3320亩。新建新开铺、星沙北等污水处理厂，城市污水处理率达80%。城市空气质量优良率保持在90%以上。建设人民满意城市，在全国文明指数测评中居省会和副省级城市第4位。被评为中国十佳休闲宜居生态城市。

　　（四）新农村建设有效改善农村生产生活条件。实施农村公路通达、电力扩容、安全饮水和环境整治四大工程，新建改造农村公路2100公里；新解决18.3万人饮水安全问题；率先全省开展山塘清淤，清理山塘6329口；新建沼气池 1.25万口；完成农村电网改造投资1.8亿元；县（市）城污水处理厂全部建成运营，6个乡镇污水处理厂投入使用，乡镇垃圾收集处置率达40 %。粮食总产量达248.9万吨，农产品加工销售收入430亿元。养殖业产值达128.8亿元，被评为全省动物疫病防控红旗市，全国首家生猪产品电子交易所挂牌运营。集体林权制度改革全面铺开，农村土地经营权流转稳步推进，发展农民专业合作组织430家。莲花镇、关山村、光明村和“两区两片”新农村示范建设成为亮点。

　　（五）综合配套改革有力推动“两型社会”建设。坚持生态优先，加强源头治理，节约集约用地，新建建筑全部执行节能标准，新增项目推行能耗审核和节能评估制度，实行排污权交易和生态资源补偿制度，对环境风险企业开展分类管理，开展节能与新能源汽车推广试点，宾馆酒店等场所停止免费提供一次性日用品。先导区建设加快推进，滨江新城、梅溪湖、岳麓山大学城等片区开发进展顺利，枫林西路等5条主干道竣工通车，“四纵八横”道路骨架基本成型。全市单位GDP能耗降低4%以上，SO2排放和COD分别减少11.08%和22.16 %。长沙县获评“中国十佳两型中小城市”；开福区青竹湖镇荣获全省第一个“两型社会示范镇”称号；长沙县松雅湖和宁乡县金洲湖国家湿地公园成功获批。

　　（六）各项事业发展推进社会全面进步。推进教育强市建设，义务教育合格学校达80.7%。实施引进国际高端人才和先进适用技术、引进储备万名优秀青年人才三年行动计划。完成“两馆一厅”主体工程，新建未成年人绿色上网场所100家，建成农家书屋138家，完成全市非物质文化遗产普查和第三次全国文物普查，又有2件作品荣获全国“五个一工程奖”。开展全民健身，成功承办第十届全国中学生运动会、举办市第七届运动会。启动医药卫生体制改革，城乡医疗卫生服务体系逐步完善，甲型H1N1流感等重大疾病得到有效控制。继续保持低生育水平，人口自然增长率为5.1‰。落实民族政策，促进宗教和睦，被评为全国民族团结进步模范集体。工会、共青团、妇联、科协、文联、残联、侨联、社科联、贸促会、红十字会、关工委、老龄委等群团组织为长沙发展做出新贡献。

　　（七）保障民生利益促进和谐长沙建设。推进创业富民，以创业带动就业，积极帮助大学毕业生就业和扶持大学生自主创业，全年新增城镇就业11.68万人，城镇登记失业率3.47%，实现零就业家庭100%动态清零。启动城镇老年居民养老保险工作，加强城乡低保工作规范化管理。参合农民住院医疗费用补偿率达36.4%，在校大学生纳入医疗保障范围，城镇居民医疗保险参保达117.9万人。强化社会治安综合治理，完善突发事件应急管理机制，建立实施“一健三联”信访工作机制，抓好国庆60周年和各项重大活动安全维稳工作。加强国家安全、国防建设和人防工作，驻长部队和民兵预备役人员在经济社会发展中发挥了积极作用。坚持标本兼治，食品安全城市建设取得良好成效。圆满完成第二次全国经济普查。4个区荣获“全国和谐社区建设示范城区”称号，我市社区建设经验在全国推广。

　　（八）政府管理创新进一步优化发展环境。开展“行政效能年”活动，推行行政审批流程再造，政务服务中心实行首席代表常驻制度，实现“一门式”办理。自觉接受人大和政协监督，认真办理人大代表议案、建议和政协委员提案。加强政府投资管理，形成从项目立项、预算控制、招投标管理到投资审计的全过程监管链条，审计核减18.4亿元。深化国有企业改革，完善国资监管体系。加强安全生产监管，较大以上安全生产事故同比下降90.9 %，安全生产示范工作在全国推广。推行全员绩效考核，建立政府雇员制度。启动政府机构改革前期工作。严格执行廉政建设八项制度，强化行政监察和审计监督，严肃查处各类案件，审计工作被评为全国先进。启动公务消费制度综合改革试点，开展行政事业单位经营性资产清查和“小金库”专项治理。统计、对台、外侨事务、法制、档案、机关事务管理、市志、气象、地震、公积金管理、移民等工作取得新成绩。

　　各位代表，我们之所以能够在逆境中砥砺奋进，实现又好又快发展，是省委、省政府和市委科学决策、正确领导的结果，是市人大、市政协加强监督、支持帮助的结果，是全市人民迎难而上、团结拼搏的结果。在此，我谨代表市人民政府，向全市各族人民，向驻长部队、武警官兵，向公安政法干警，向各民主党派、工商联、人民团体和各界人士，向所有关心支持长沙发展的朋友们，表示衷心的感谢！

　　二、2010年形势与发展思路

　　2010年是实施“十一五”规划的最后一年，也是启动“十二五”规划的奠基之年。总体看，世界经济止跌回稳，我国经济回升向好，今年经济发展环境将好于去年。但经济复苏将是一个缓慢曲折的过程，经济社会发展仍然存在一些薄弱环节，面临许多困难和挑战。

　　一是转变发展方式的任务十分艰巨。国际金融危机对我国经济的冲击，表面上是对经济增长速度的冲击，实际上是对经济发展方式的冲击。中央应对危机的“一揽子计划”取得了阶段性成效，但是，外向型经济对出口的依赖与外需不振、扩大消费需求与居民消费能力有限等深层次矛盾还未得到很好解决，经济增长的基础还不稳固，发展的可持续性受到制约，发展方式实现根本转变需要付出更加艰辛的努力。

　　二是影响经济增长的不确定因素增多。短期内，出口、消费难以为经济增长提供强劲动力，投资拉动仍是经济增长的主动力，但中央在保持宏观调控政策基本取向不变的同时，将增强调控的灵活性和针对性，预期投资方向会有所改变，信贷投放力度、项目审批速度、土地供应强度等有可能偏紧，对我市新建项目的审批、续建项目的融资、项目建设的供地都会产生较大的影响。

　　三是资源环境约束压力更加突出。节能减排作为约束性指标，纳入国民经济和社会发展中长期规划；人民群众对干净的水、新鲜的空气、优美的环境等方面的要求越来越高。由此带来企业准入门槛抬高，产品生产成本增加，环保投入的财政压力增大。尽管我们连续三年超额完成了节能降耗任务，但我市节能减排、环境保护的任务还相当艰巨，既要偿还环保历史欠账，又要做好当前环境治理工作。同时，低碳发展方式尚未形成，经济发展和人口增长又带来新的环境压力，资源环境的约束日益增强。

　　四是经济结构调整优化升级难度较大。我市产业结构调整取得了可喜进展，但发展方式仍然比较粗放，工业增长过分依赖于单个产业，抗风险能力较弱；企业自主创新能力不强，战略性新兴产业刚刚起步。从全国看，不少行业生产能力相对过剩，即使一些新兴产业也可能因为各地竞相发展产生新的产能过剩问题。依靠核心技术促进产业升级、发展高端产品增强核心竞争力、实现产业多元化提高抗风险能力、培育战略性新兴产业促进经济结构优化，需要相当长的时间。

　　五是民生保障与和谐社会建设任务繁重。近年来，长沙政治社会稳定，人民安居乐业。和全国一样，我市仍处于社会矛盾凸显期，一些历史遗留问题和发展中的新矛盾、新问题相互交织。车辆快速增长，城区交通拥堵的矛盾比较突出；部分群众生活比较困难，社保、教育、医疗、住房等问题还没有得到根本解决；普通高校毕业生和城镇新成长劳动力不断增多，就业压力持续增大；个别重大刑事犯罪案件直接危害人民群众生命财产安全，维护稳定任务艰巨。同时，政府管理水平与繁重的社会管理任务不相适应，政府服务需要进一步改善，行政效能有待进一步提升，发展环境仍需进一步优化。

　　各位代表，我们面对的困难和挑战既有共性的，也有个性的，必须高度重视，科学应对。同时，我们也要充分认识到，长沙具有许多独特的优势。作为中部省会城市，我们具有成本优势、土地优势，武广高铁开通和沪昆高铁、全省30多条高速公路建设，进一步强化了我们的地理优势。我们还拥有人才智力优势和生态环境优势，可以吸引高端人才和高端产业。同时，我们的文化产业和消费知名度己形成独特优势。特别是作为国家“两型社会”建设综合配套改革试验区，在发展环保产业、低碳经济方面，我们与发达地区和先进城市站在同一起跑线上，走在了全国的前面。只要把握好这个机遇，加大科技投入、智力引进、技术创新，未来竞争将对我们更加有利。我们要把长沙放在全省、全国乃至全球发展中来谋划和定位，树立国际视野，增强战略思维，扬长避短，发挥优势，保持经济社会平稳快速发展的良好势头。

　　基于上述分析，今年政府工作的总体要求是：全面贯彻党的十七届四中全会和中央、省、市委经济工作会议精神，认真落实科学发展观，深入实施“一化三基”战略，切实转变发展方式，突出产业结构调整，突出重大项目建设，突出城乡一体化发展，突出消费需求拉动，突出“两型社会”建设，突出改善民生，大力推进政府精细化管理，努力实现又好又快、率先发展。

　　今年政府工作的基本思路是：发挥优势，充分利用长沙的地理、成本、人才、智力和生态环境优势以及后发优势，抢抓发展机遇，推动科学发展；保持投资，突出抓好关系长远发展的重大项目建设和竣工投产，激活民间投资，保持投资对经济增长的拉动作用；扩大消费，坚持本地消费和外来消费并重，更加注重吸引外来消费，以良好的生态环境和消费环境吸引周边的消费人群，建设区域消费中心；升级产业，加强产学研金联合，引导和支持企业自主创新，改造提升传统产业，坚决淘汰落后产能，推动结构调整优化升级；拓展领域，促进产业多元化，在做大做强优势产业的同时，积极培育有市场、有基础、有优势的战略性新兴产业，突出发展低碳经济，增强可持续发展和抗风险能力；普惠民生，落实惠民政策，解决群众生产生活困难，让经济发展的成果和老百姓的切身感受相匹配，实现经济发展的速度和老百姓的满意度同步上升；提高品质，坚持以质取胜，提升城市品位和居民生活品质，打造现代宜居城市，以品质吸引人才、吸引投资、吸引消费；注重基础，我们面对的矛盾既有当前的，更有长远的，必须更加重视应对长远挑战，准确把握当前和长远的平衡点，从最基础的工作做起，避免急功近利行为，加强基础设施和产业项目建设，增强发展后劲，提高长远竞争力。

　　今年经济社会发展的主要预期目标是：地区生产总值增长12%；地方财政收入增长13%；全社会固定资产投资增长20%；社会消费品零售总额增长16%；单位地区生产总值综合能耗下降4%，主要污染物排放总量减少4%；城乡居民人均可支配收入分别增长7%、9%；城镇登记失业率在4.5%以内；人口自然增长率在5.5‰以内；居民消费价格指数在省会城市平均水平以下。

　　在具体实践中，需要更加重视以下几个方面：

　　一是高度重视城市空间发展战略。选准空间发展战略重点，往往事半而功倍。在坚持全面发展的同时，更加突出发展重点，强化中心城市功能。加快浏阳西进、宁乡东扩，加速长沙、望城两县与主城区对接融合，城区向城市中心相向发展，集中资源，凝聚人气，实现可持续发展和效益最大化。先导区近期着重抓好短时间内能够见效的若干片区，建一片，成一片，享受一片，带动一片。

　　二是高度重视高铁建设带来的战略机遇。武广高铁开通和沪昆高铁建设开启长沙与珠三角、长三角的快速通道，并产生强大的虹吸效应，吸聚沿线城市资源。科学谋划武广片区发展，选准产业方向，完善服务配套设施，加大建设力度，进一步巩固长沙服务全省、服务中部以至服务华东、华南、西南地区的地位。

　　三是高度重视发挥基础设施的效益。城市道路等基础设施建设投入大、周期长，建设重点和时序对城市建设和财政收支都有很大影响。应更加重视科学决策，先建什么、后建什么，建在什么地方，都要经过详细论证，把投入放在最需要、最紧迫的地方，最大限度发挥投资效益，决不搞“晒太阳工程”。

　　四是高度重视生态环境保护和城市品质建设。优良的城市品质和良好的生态环境是宜居城市的重要标志。突出提高城市国际化水平，增强对外吸引力和影响力；突出城市生态化，彰显山水洲城品质；突出细节雕琢，使每一项工程都匠心独具；突出历史文化、历史街区、城市风貌保护，传承城市文脉。

　　五是高度重视群众的切实感受。在发展经济的同时更加注重群众需要，分析群众最关心什么、最需要什么，最大程度满足不同层次群众的合理需求，以此来确定工作重点。认真研究解决居民就业、安居、社会保障等问题，使经济发展的过程成为群众享受成果、得到实惠的过程，让群众切实感受到发展带来的好处。

　　六是高度重视和关心基层。基层组织处在困难、矛盾的最前沿，条件艰苦、待遇清苦、工作辛苦。坚持重心下移，继续简政放权，加强对基层工作的指导，关注基层、关心基层、爱护基层、支持基层，多排忧解难，少增加负担，切实改善基层待遇和工作条件，让基层干部感到服务基层有甜头、扎根基层有盼头。

　　三、2010年工作重点

　　推进城乡一体化发展是今年和今后一段时期的一项重大战略任务。我们要按照市委《推进城乡一体化发展工作纲要》的统一部署，有序推进城乡规划、基础设施、公共服务、产业发展、生态环境和管理体制一体化，加快创业之都、宜居城市、幸福家园建设。今年重点抓好以下几个方面的工作：

　　（一）推进产业结构调整和优化升级。抢占后金融危机时代的发展制高点，关键在于加快转变经济发展方式。利用成本优势发展传统产业，利用人才智力优势发展战略性新兴产业，利用地理优势承接发达地区产业转移。做大做强优势产业集群。以园区为载体，以工程机械、汽车制造及零部件、食品烟草、新材料等大型企业为龙头，培育配套中小企业群体，延长产业链，力争千亿产业集群零的突破。抓好园区项目储备和土地储备，主动承接产业转移，促进产业集聚发展。加快文化产业发展，建设文化创意产业集聚区。扶持发展战略性新兴产业。推进以中电软件园为核心的电子信息产业、以浏阳生物医药园为核心的生物医药产业、以光伏太阳能为核心的新能源产业、以远大住宅工业为核心的新材料产业、以武广片区为核心的高铁经济发展。支持高新区建设创新型科技园区，推进经开区国家新型工业化产业示范基地建设。大力发展现代服务业。突出壮大生产性服务业，重点发展现代金融、现代物流、电子商务、咨询中介等产业，加强服务外包基地和芙蓉中央商务区建设。积极发展生活性服务业，引导发展便民服务和市政公用事业。加快发展现代农业。积极发展城郊型、生态型农业，稳定发展县域经济。突出十大农业主导产业，稳步提高粮食综合生产能力，扶持农产品精深加工龙头企业发展，全市农产品加工销售收入增长18%以上。推进农业标准化生产，推行农产品质量安全管理和市场准入制度，加强动物疫病防控。扶持发展农民专业合作组织，发挥供销社、信用社的服务功能，完善农村社会化服务体系。

　　（二）推进重大项目建设。继续实施重大项目带动战略，保持投资强度。全年计划重点项目485个，完成投资1333亿元。拓展融资渠道。完善和落实鼓励民间投资的各项政策，激活民间投资。进一步创新招商方式，优化招商环境，全年引进外资22亿美元，引进市外境内资金635亿元。发挥城建投、先导投等融资平台作用，启动地方产权交易市场建设，支持企业上市融资，扩大小额贷款公司试点。开展农村合作金融综合改革，创造条件发行中小企业集合债券和信托产品。优化投资结构。坚持以投资促进经济结构调整，加大重大产业项目、重大基础设施和重大民生项目建设投入，力争产业投资占全社会投资50%以上，工业投资增长25 %以上。突出重大项目建设。完成60号令拆迁扫尾工作，以拆迁推进项目建设。加快广汽菲亚特、比亚迪等重大项目建设，尽快形成中国汽车工业新的板块。加快地铁2号线、营盘路过江隧道、湘江综合枢纽等重大工程建设，完成南北货站迁建。

　　（三）推进城乡一体化。落实强农惠农政策，促进城乡资源优化配置，实现以城带乡、城乡融合发展。加快城乡建设互动对接。促进城乡基础设施同网规划、同步建设，抓好浏大、开元东路等干线公路改造建设，加快长浏、长韶娄等高速公路建设，完善城乡路网。加强城乡结合部规划管理，支持县（市）城提高综合承载能力，调整优化城镇布局，重点扶持20个中心镇和50个中心村的规划建设。继续推进农村“四大工程”建设。新增硬化通村公路1500公里；全面完成农村电网改造提质；新解决45万农村人口安全饮水问题，集中力量用三年时间完成水库除险保安和骨干山塘清淤工作；新建沼气池1万口；加大农村固体垃圾处理力度，建成乡镇垃圾转运站19个、村级垃圾收集站300个，建成乡镇污水处理厂12个，完成乡镇集镇环境综合整治20个。突出解决农村四对矛盾。着力解决农业比较效益相对较低和农民收入亟待提高之间的矛盾，农民就业转移与城市就业岗位有限之间的矛盾，现代农业要求规模经营与农村土地个人承包之间的矛盾，新农村建设需要高素质农民与农村人口素质偏低之间的矛盾，加快农村人口向城镇转移，推动农业产业化，增加农民收入。

　　（四）推进区域消费中心建设。充分利用高速铁路、高速公路、航空、水运等综合交通优势和周边消费需求，创造更好的城市品质、生态环境，提高消费吸引力。扩大消费需求。提高城乡劳动者收入水平，增强城乡居民消费能力。落实家电、汽车下乡及以旧换新等政策，鼓励发展消费信贷，扩大居民消费。支持外经外贸企业出口，扩大外部需求。培育消费热点。启动电信、电脑、电视“三网融合”，促进信息消费。发展会展经济，办好重大节会。加快岳麓山、灰汤温泉、铜官窑等重点景区建设，打造一批旅游精品项目，发展生态旅游，扩大旅游消费，将旅游业打造成为战略性支柱产业，实现旅游总收入400亿元以上。优化消费环境。加强五一商圈、黄兴路步行街、东塘商业中心等特色商业街区建设，科学规划建设先导区商业网点，打造商业地标和大型商业综合体。推进农村商品交易市场、商品配送中心、“万村千乡”市场工程建设，继续推进农贸市场提质改造。加强食品生产经营秩序整治，推进食品安全城市建设。推进征信和信用体系建设，营造便利、安全、放心的消费环境。着力构建长株潭“半小时消费圈”、“3+5”城市群“一小时消费圈”、省外周边城市“两小时消费圈”，把长沙打造成为具有全国影响力的区域消费中心。

　　（五）推进现代宜居城市建设。坚持精细管理城市规划、建设和运行，完善城市功能，提高城市品位。注重规划引导调控。加快城市总体规划修编，统筹各类规划的编制，注重要素聚合，增强规划的科学性。加强对各县（市）的规划指导和管理，构建城乡一体的规划国土管理新体制。落实色彩、绿线规划，推进规划进社区工作，强化规划实施的社会监督。加快中心城区建设。按照“打通出口、畅通城区、联接两岸、提升品质”的思路，重点推进中山西路、南湖等片区建设，启动地铁1号线、南湖路过江隧道、福元路湘江大桥、橘子洲大桥扩建、湘府路大桥、餐厨垃圾处理中心等项目建设；进行黄兴北路、东二环改造；加强城区停车场建设。不断改善居住环境。扩大城市绿地规模，实施湘江两岸亮化工程，新建社区公园20个、站厕30套，完成5条景观街建设，加快15个困难企业小区环境综合整治。完成城区截污口改造和排水堵点疏浚，力争城区污水全截污、全收集、全处理。巩固渣土、扬尘、噪音和机动车尾气污染治理成果，城市空气质量优良率保持90 %以上。提升城市管理水平。城市重要节点、主要干道往往体现城市的气质和形象，要重点加强整治和管理。全面推行网格化管理和开放式老旧居民社区物业化服务，加强物业服务企业规范管理。坚持公交优先，实施交通疏导工程三期。继续加强违章广告和门店招牌整治，坚决遏制新的违法违章建筑产生。夯实文明创建基础工作，不断提高市民素质，抓好各项创建项目达标，推进人民满意城市建设。

　　（六）推进“两型社会”建设。坚持重点突破、全面推进，着力在关键领域和重点环节先行先试、取得实效。加速大河西先导区建设。完成岳麓大道西延拓改、坪塘大道等6条道路建设，启动洋湖大道、黄桥大道二期建设，形成核心区道路基本框架。推进滨江新城、梅溪湖、岳麓山大学城三大片区开发，全面完成岳麓山风景名胜区环境整治，基本完成坪塘老工业基地退出。着力推进节能减排。全面实施环保三年行动计划，突出工业污染、农村面源污染、城乡垃圾治理。实施生猪标准化规模养殖，城区退出畜禽养殖业，完成500头以上生猪规模养殖场污染治理。加强湘江流域长沙段饮用水源保护和浏阳河、沩江等支流水质环境治理，完善城乡环境质量监测网络。继续抓好重点领域、重点企业和重点建筑节能节水改造，推广绿色建筑，推进节约型城市和国家节水型示范城市建设。加大重点领域改革创新。实施新增项目能耗审核制度。推进污水处理、垃圾处理收费制度改革，开展生态补偿试点，实行市级生态公益林补偿制度，扩大环境资源交易规模。推进土地综合整治、城乡建设用地增减挂钩、“先征后转”，实施耕地占用补偿制度，加大闲置用地处置力度。加强林地承包经营权管理，规范农村土地流转，稳步推进农村建设用地使用权交易。探索实行城乡统一的户籍管理制度，使在城镇就业和居住的农民有序转变为城镇居民。力争完成国有企业改革。

　　（七）推进民生事业发展。突出抓好省市为民办实事工程，着力解决一批群众最关心、最直接、最迫切的民生问题，不断提高民生保障水平。完善社会保障体系。制定城乡居民基本养老办法，完善被征地农民社会保障制度，基本养老、基本医疗、失业、工伤和生育保险分别新增参保16.8万、2.5万、4.38万、4万和2.3万人。加强社会救助、社会福利、慈善和红十字会工作，加大扶贫帮困力度。推进新型农村养老保险试点，提质改造乡镇敬老院6 所，每个城市社区建设1个居家养老服务站。坚持创业带动就业。继续实施创业富民工程，全面落实税收优惠、小额担保贷款、资金补贴等政策，鼓励和扶持更多劳动者自主创业。完善就业信息平台，加强就业援助，健全城乡统一的人力资源市场，完成职业技能培训13万人次，全年新增就业岗位 8.7万个，保持“零就业家庭”动态清零。大力扶持民营企业发展，着力解决中小企业融资难问题，促进中小企业发展，有效吸纳就业。突出解决居民住房问题。加大保障性安居工程建设，进一步规范经济适用房货币补贴发放，开展“城中村”改造试点，推进棚户区改造，力争完成棚户区拆迁200万平方米，新建（筹集）廉租房6000套。改造农村危房2000户。住房是群众花钱最多、关注最多的问题，住有所居是每个人的梦想，政府要千方百计帮助群众圆梦。

　　（八）推进社会建设。把加强社会建设作为扩大内需的重要举措。加快创新型城市建设。加强知识产权保护，加快实施重大科技专项，引导支持企业自主研发，完成技改投入660亿元，科技成果转化率提高2个百分点，高新技术产业总产值增长25%。加强人才队伍建设。注重培育本地人才与引进外来人才相结合，继续实施引进国际高端人才和储备万名优秀青年人才三年行动计划，以人才结构的转型优化促进发展方式转变。建设教育强市。继续实施中小学校舍安全工程，完成职教基地建设，推动城乡教育资源均衡配置，促进各类教育协调发展，突出解决好困难家庭子女上学问题。进一步加强未成年人思想道德建设。强化素质教育，减轻学生和教师负担，让孩子们快乐学习，健康成长。实施文化强市战略。加强公共文化服务体系建设，加快青少年宫等文化站场（馆）建设，推进农家书屋工程。完成文化市场综合执法改革和市歌舞剧院改制。加强历史文化街区（巷、古镇）、历史文化遗存和非物质文化遗产保护和利用。加强健康城市建设。加强公共卫生服务体系建设，实施医药卫生体制改革，加强重大传染疾病预防控制，稳妥推进参合农民门诊统筹补偿、宁乡基层医疗卫生综合配套改革试点。开展全民健身运动，组团参加省第十一届运动会。稳定低生育水平，提高出生人口质量。抓好第六次全国人口普查工作。建设和谐长沙。健全信访工作机制，畅通群众诉求渠道；加强社会治安综合治理，完善军警民联防联治和应急管理体系，推进社会矛盾化解和社会管理创新，维护社会大局稳定。发挥宗教界的积极作用，促进民族团结进步。严格落实安全生产责任制度，抓好重点领域生产安全，严防重特大事故发生。增强国家安全和全民国防意识，加强民兵预备役建设，争创全国“双拥模范城”五连冠。

　　在做好以上工作的同时，按照体现特色和发挥优势相结合、当前发展与长远发展相结合的要求，广泛调查研究，深入系统分析“十二五”全市经济社会发展的全局性重大问题，问计于民，问计于专家，集思广益，科学编制“十二五”总体规划和各专项规划，为长沙科学发展、率先发展提供行动纲领。

　　四、大力推进政府精细化管理

　　政府服务质量和管理效益对一个地方的经济社会发展有着至关重要的影响。以新一轮政府机构改革为契机，推进政府精细化管理，实现用同样的钱干出更多的事，同样的人为群众提供更好的服务，建设人民满意政府。

　　（一）坚持科学决策。决策带来的效益是最大的效益，决策产生的失误是最大的失误，必须慎之又慎。推行开门决策。继续实行政府常务会议、市长办公会议市民代表旁听制度，并逐步向各区县（市）和各部门延伸。继续开展市政府常务会议电视、电台、网络同步直播。实行民主决策。进一步完善领导、专家和群众相结合的决策机制，完善重大事项专家咨询、社会稳定风险评估、公示听证制度，所有重大决策都要深入调查研究，广泛听取意见，通过充分论证，集体讨论决定。规范决策程序。优化政府重大决策规则和法定程序，保证决策的科学性和正确性。特别是重大投资决策，要严格立项审查，严格招投标管理，优化建设方案，控制投资规模，实现投资效益最大化。通过科学决策，争取不犯错误，尽量少犯错误，特别是不犯不可纠正、无法弥补的错误，使政府决策经得起历史和实践的检验。

　　（二）提升工作质量。强化“把每一件简单的事做好就是不简单”的理念，提高政府工作水平。细化量化目标责任。按照可量化、可操作的原则，科学制定涵盖政府管理服务全过程、全方位的流程规范和技术标准，逐级为每一个岗位制定科学具体的职位说明书，细化目标、量化责任，使政府管理成为目标明确、责任具体、能进行量化考核和责任追溯的过程。工程建设精益求精。随着一大批项目开工建设，加强工程管理、确保建设质量比以往任何时候都更为重要。坚持全寿命周期成本理念，实施工程建设质量精细化管理，精心规划设计，规范建设标准，精细施工建设，尽量减少后期维护成本，建设高品质耐久性工程。财政收支精打细算。加强税收征管，堵塞征管漏洞，做到应征尽征。创新国有资产监管方式，规范公共资源交易管理，发挥国有资本的带动作用，实现国有资产保值增值。推进预算公开，加强财政资金监管，落实政府性投资项目资金“双控”管理，加强重大投资项目审计，管好用好每一分钱。完善政府采购制度，规范公务消费行为，确保全年公用经费零增长。加强公务员队伍建设。开展公务员岗位培训，提高公务员专业素质。开展轮岗交流，增强队伍活力。崇尚朴实无华的作风，从群众身边的事干起，从群众不满意的地方改起，多为群众办实事。

　　（三）规范行政行为。严格依法行政，建设法治政府，运用法律和制度规范行政程序和行政行为，促进行政权力公开、公平、公正运行。坚持制度管人管事。建设管用、完善、精细的政府管理服务制度体系，加强制度的合法性审查，依靠制度规范和加强政府管理，做到每项制度有人执行落实，违反制度有人承担责任，提高政府执行力和公信力。规范优化行政审批。继续清理和规范行政审批，加强电子政务建设，完善行政许可项目在线审批系统和电子监察系统，对行政审批行为实行全过程监控。继续清理和规范行政收费，健全为企业、项目服务机制，积极为企业和基层排忧解难，优化发展环境。严格行政过错责任追究。实行行政执行跟踪、责任追究等制度，健全信息反馈、绩效评估和纠偏机制，建立责任追溯体系，严格行政问责，认真查处群众反映强烈的突出问题。

　　（四）强化行政监督。坚持组织监督与群众监督相结合、内部监督与外部监督相结合，确保行政权力运行公开透明。实行阳光监督。自觉接受人大和政协的监督，及时办理人大代表议案、建议和政协委员提案，认真听取和采纳各民主党派、工商联、人民团体和社会各界人士的意见，主动接受新闻媒体和社会公众监督。强化行政监察和审计监督，建立监察、财政、人事、审计、法制等行政监督部门协调沟通机制。推进政务公开。加强政务中心建设，全市所有乡镇（街道）建立政务中心。落实《政府信息公开条例》，实行公共政策、部门责任“白皮书”制度，定期向社会公布。加强廉政建设。更加注重反腐倡廉，推进预防和惩治腐败体系建设，坚决纠正损害群众利益的不正之风，树立为民、务实、清廉的政府形象，赢得人民的信任和支持。

　　各位代表！新的形势对我们提出了更高的要求，我们必须自我加压，负重奋进，追求卓越，做一项工作，留一个亮点，创一个精品，不辜负党和人民的期望与重托。让我们在市委的坚强领导下，坚定信心，锐意进取，为率先基本实现全面小康，创造更加美好的未来而努力奋斗!
